# Imagery and motor learning: a special issue on the neurocognitive mechanisms of imagery and imagery practice of motor actions

**DOI:** 10.1007/s00426-024-01982-5

**Published:** 2024-06-28

**Authors:** Cornelia Frank, Aymeric Guillot, Stefan Vogt

**Affiliations:** 1 Sports and Movement Research Group, Department of Sports and Movement Science, School of Educational and Cultural Studies, Osnabrück University, Jahnstraße 75, 49080 Osnabrück, Germany; 2 Inter-university Laboratory of Human Movement Sciences, Universite Lyon 1, UCBL-Lyon 1, Villeurbanne, 7424, F-69622 UR France; 3 Department of Psychology, Lancaster University, Lancaster, LA1 4YF UK

## Abstract

Human beings are able to imagine actions with the aim to change movement coordination and to learn particular movements. Meta-analyses to date have shown that when individuals systematically engage in imagery of a motor action without overt behavior this can improve motor performance and facilitate motor learning. Despite a considerable body of research in neuroscience, psychology, and sport science, however, there is at present no consensus on the neurocognitive mechanisms of imagery, and the mechanisms that lead to learning via imagined action are still being debated. In particular, the differences between imagined and overt action, and respective learning effects, remain to be fully explained. The present collection of manuscripts is a result of compiling both theoretical advances in the field of motor control and motor learning and those in imagery research to better understand imagery and learning. It is structured alongside five position papers from leading experts in the field, each of which is followed by a series of short commentaries written by experts from various disciplines. This collection demonstrates (a) that conceptualizations of imagery are manifold, vary highly and depend on the perspective chosen, (b) that existing approaches to the neurocognitive mechanisms of imagery and imagery practice of motor actions draw on distinct motor control and learning perspectives, (c) that perspectives from the wider fields of motor control and learning stimulate new approaches to explain imagery and imagery practice, (d) and that future research is needed to investigate and compare different perspectives and conceptualizations of the neurocognitive mechanisms of imagery and imagery practice of motor actions.

One of many fascinating capacities of human beings is the ability to imagine actions and, what is even more intriguing, the fact that we can improve our performance and learn by way of imagining actions. Imagery in sport can be characterized as a multisensory, but vicarious experience, a (re-) creation of experience in the absence of the actual sensory stimuli (Annett, 1995; Farah, 1984; Lacey & Lawson, 2013; Morris et al., 2005). In contrast to overt action, imagined action (i.e., motor imagery, mental imagery of action, action imagery, effect imagery) relates to the imagery of one’s own action without any overt behavior (Jeannerod, 1995; Munzert & Zentgraf, 2009). Along these lines, imagery practice (i.e., mental practice; imagery training; motor imagery practice; action imagery practice) denotes the imagery of an action in a systematic and repetitive manner, while physical practice relates to the systematic and repetitive use of overt action.

Syntheses of evidence to date have shown that when humans systematically engage in imagery of a motor action without overt behavior this can improve motor performance and facilitate motor learning (Driskell et al., 1994; Simonsmeier et al., 2020; Toth et al., 2020). Although the mechanisms of imagery and imagery practice of motor actions have long been discussed alongside of numerous theories (e.g., Annett, 1995, 1996; Jacobson, 1931; Jeannerod, 1994, 1995, 2001; Sackett, 1934; Heuer, 1985, 1989; Schack, 2006), and despite a considerable body of research in neuroscience, psychology, and sport science, the neurocognitive mechanisms that lead to learning via imagined action are still being debated (Frank, 2014; Glover & Baran, 2017; Kraeutner, 2019; Moran & O’Shea, 2020). To date, a synopsis of theories and related findings in the field of imagery and imagery practice is lacking. The aim of this collection is to provide a starting point to further developing imagery theory, by drawing on existing approaches and providing new ideas that may contribute to better understand how imagery and learning by way of imagery work, and to be better able to tailor imagery practice and design effective imagery interventions to the specific needs of individuals in performance and learning settings such as sport, rehabilitation, work and physical education.

## Starting point

In the last decade, there has been an explosion of interest among human movement scientists, cognitive psychologists and neuroscientists towards acknowledging and studying the differences between imagined and overt action, and towards advancing theory development in the field (Frank, 2014; Glover & Baran, 2017; Kraeutner, 2019; Moran & O’Shea, 2020). Interestingly, recent theoretical advances made in the wider fields of motor control and learning have not yet been sufficiently considered in imagery research. Likewise, and despite the fact that imagery contributes to human performance and learning, motor control and learning theories have rarely addressed imagery and respective effects on performance and learning. To compile both the theoretical advances in the field of motor control and motor learning and those in imagery research might thus help to understand the underlying mechanisms mediating imagery practice in greater detail.

To address this gap, we organized an online-workshop in November 2020, hosted by the Center of Interdisciplinary Research (ZiF: Zentrum für interdisziplinäre Forschung) at Bielefeld University. The aim of the workshop was to inspire, for the first time, future research on imagery practice and its mechanisms linking recent theoretical advances in imagery research to those made in the wider fields of motor control and learning. To this end, the workshop brought together experts in motor control and motor learning with experts in motor imagery research from cognitive psychology, neuroscience, and sport science to explore current developments and their implications for understanding imagery practice. Over the course of three days, Patric Bach, Shaun Boe, Cornelia Frank, Scott Glover, Aymeric Guillot, Robert Hardwick, Mathias Hegele, Nicola Hodges, Wilfried Kunde, Britta Krüger, Chris Miall, Martina Rieger, David Rosenbaum, and Stefan Vogt discussed various perspectives with the aim to jointly develop integrative, specific, and testable models explaining the nature of learning effects of imagery practice.

The present compilation of manuscripts is both an outcome and a continuation of the workshop: Five position papers originated from discussions during the workshop, while commentaries were written by experts from various disciplines and target one of the five position papers published. The special issue is structured accordingly: A series of short commentaries follows each of the five position papers.

Altogether, this collection reveals (a) that conceptualizations of imagery are manifold, vary highly and depend on the perspective chosen, (b) that existing approaches to the neurocognitive mechanisms of imagery and imagery practice of motor actions draw on distinct motor control and learning perspectives, (c) that perspectives from the wider fields of motor control and learning stimulate new approaches to explain imagery and imagery practice, (d) and that future research is needed to investigate and compare different perspectives and conceptualizations in order to advance the neurocognitive mechanisms of imagery and imagery practice of motor actions.

## Five perspectives on the neurocognitive mechanisms of motor imagery practice

The five position papers originating from the workshop all aim at explaining imagery and/or imagery practice of motor actions and related simulation phenomena, while providing five unique points of view and key aspects of current debates about the mechanisms of imagery and imagery practice of motor actions. Three of the five position papers (Bach et al., 2022; Krüger et al., 2022; Rieger et al., 2023) focus on imagery of motor actions (i.e., the actual state of imagining a motor action), while two papers (Eaves et al., 2022; Frank et al., 2023) emphasize imagery practice and the learning of motor actions by way of imagery (i.e., changes caused by imagining motor actions in a repetitive and systematic manner).

Patric Bach, Cornelia Frank, and Wilfried Kunde (2022) offer a new conceptualization of imagery in their position paper “*Why motor imagery is not really motoric: towards a re‑conceptualization in terms of effect‑based action control*”. Contrary to the current, widespread notion of motor imagery, in this provocative article, the authors argue against the well-established notion that imagery is primarily motoric. Instead, they feature the ideomotor view that imagery can largely be explained by effect-based accounts of action. The central idea of this re-conceptualization is that imagined and overt action are linked because action planning is primarily imaginistic, being characterized by the perceptual effects we want to achieve by way of our actions.

Along these lines, Cornelia Frank, Sarah Kraeutner, Martina Rieger, and Shaun Boe (2023) delineate perceptual-cognitive scaffolding as an explanation of consistently reported learning effects in their position paper “*Learning motor actions via imagery - perceptual or motor learning?*”. They argue that, if we assume a close linkage between anticipated action effects and imagery, learning by way of imagined action is possible because repeated anticipation of action effects during imagined action leads to scaffolding of (quasi-)action effects, which, in turn, helps to guide future action. From this ideomotor view, but with a focus on learning by way of imagined action, the authors account for differences between imagined and overt action, and learning by way of imagined and overt action, and demonstrate that perceptual-cognitive scaffolding is well-suited to explain what is being learnt during imagery practice, depending on various factors such as task, skill level, and instruction.

Martina Rieger, Shaun Boe, Tony Ingram, Victoria Bart, and Stephan Dahm provide “*A theoretical perspective on action consequences in action imagery: Internal prediction as an essential mechanism to detect errors*” by reviewing evidence on simulated consequences of action during imagery, and errors in imagined actions, in particular. Drawing on ideomotor accounts and on feedback mechanisms in (overt) action control, the authors argue for the existence of action consequences in imagined action, and the role that errors and their detection during imagery play in order to produce adequate feedback and thus facilitate error correction and related learning processes. Their model sketches how such a simulation may take place in action imagery, and how action consequences can be predicted by forward models during action imagery, being inhibited at some point in order to not become an overtly visible action.

Britta Krüger, Mathias Hegele and Martina Rieger (2022) widen the narrow focus of motor imagery and conceptualize imagery as an (imagined) multisensory action in their position paper “*The multisensory nature of human action imagery*”. As such, they trace action imagery as a multisensory experience including different modalities, and varying in its vividness and related effects. On this basis, Krüger and colleagues (2022) focus on the sensory quality of imagery and discuss how imagery instruction and experience, for instance, may cause differences in sensory impressions of action images, closing with related open questions to stimulate future research directions.

Finally, Daniel Eaves, Nicola Hodges, Gavin Buckingham, Giovanni Buccino and Stefan Vogt (2022) discuss various routes of “*Enhancing motor imagery practice using synchronous action observation*”, thus widening the scope from pure imagery to include observational learning, the other major form of non-physically practicing a motor skill. The authors provide an up-to-date review on the use of action observation together with motor imagery (AO + MI), and focus on the benefits of synchronously observing an action and imagining oneself performing this (or a different) coordinated action at different stages of practice. Ideas on the design of practice schedules and their theoretical relevance are being developed, such as model variation or variability of practice during AO + MI. The authors furthermore discuss coordinative cases of AO + MI where observed and imagined actions are different and coordinated with each other, they discuss whether two action simulations are run in parallel during AO + MI, and they close with a short outlook on applications of AO + MI in motor rehabilitation.

Following each of the five position papers, commentaries critically discuss distinct aspects proposed in the respective position paper, question points raised, delineate a position arguing against, and/or add aspects that have not yet been considered. We are delighted that many colleagues from psychology, neuroscience, movement and sport science provide fresh perspectives on the neurocognitive mechanisms of imagery and imagery practice/ learning from their perspectives, not only from the area of imagery, but also from the wider fields of motor/ action control and learning, opening up new avenues for imagery and learning.

## Advancing the neurocognitive mechanisms of imagery practice

Where do we go from here, from the variety of conceptualizations, from current approaches described and ideas for potential future approaches sketched, from increasingly intertwining imagery and motor control/ action control and learning research?

In order to determine and formalize the exact relation between cognitive processes and movement, here between imagined action and action control as well as the learning of a motor action, specific mechanistic theories are needed to determine the functioning of imagery of motor actions and learning through imagery. Alongside of the claim that theories in cognitive science are only truly mechanistic if they include both structures and processes, together with their relations (Bechtel, 2008; Hommel, 2019), future models and theories relating imagery to action control and learning may try and include all three aspects, namely which structures are involved, which processes takes place and how the two interrelate with one another: “Translated into cognitive (neuro)science, a good mechanistic theory would thus consist of a clear specification of its components, such as the codes or representations of the relevant informational units, and of the organization of these components, including the processes operating on them (Bechtel, 2008, 2009). In other words, mechanistic theories need to explain how structures relate to processes, and vice versa” (Hommel, 2019, p. 2). We therefore advocate to go more mechanistic, and to try and formalize the relation between imagery and performance/ motor learning further: “Cognitive and neurocognitive theory [here: Imagery theory] thus needs to become more ambitious in terms of aims and mechanistic detail – irrespective of whether the explanatory language is functional, neural or computational in nature” (Hommel, 2019, p. 9).

We hope that this Special Issue will not only contribute to further develop imagery theory, but will also help to accentuate the relationship between imagery / motor control researchers and practitioners, and thereby contribute to reconcile these two worlds. We thus hope that more and more colleagues will join our challenge to build models that specify both structures and processes together with their relations and to rigorously test resulting hypotheses in future research. Innovative research questions bridging different disciplines, research areas, theories, basic/ applied research will stimulate future research, and will help to find more specific answers in order to further approach the mechanisms why imagery is effective in learning motor actions, and thus to advance imagery theory.

## Data Availability

No datasets were generated or analysed during the current study.
